# Predictors of Residual Stone Fragments After Ureteroscopic Lithotripsy for Upper and Mid-ureteric Calculi: A Prospective Observational Study

**DOI:** 10.7759/cureus.112454

**Published:** 2026-07-11

**Authors:** Vignesh Vivekanandam, Anurag Singla, Hemant Goel, Ashish Varshney, Nishok Raj

**Affiliations:** 1 Urology, Atal Bihari Vajpayee Institute of Medical Sciences and Dr. Ram Manohar Lohia Hospital, Delhi, IND

**Keywords:** hounsfield units, lithotripsy, stone-free rate, ureteroscopy, urinary calculi

## Abstract

Introduction

Residual stone fragments following ureteroscopic lithotripsy (URSL) contribute to stone recurrence, UTI, and the need for repeat procedures. Identifying preoperative predictors of incomplete stone clearance is essential for optimizing surgical planning and patient counselling. We aimed to evaluate clinical, stone-related, and operative predictors of residual fragments after URSL for upper and mid-ureteric calculi.

Methods

This prospective observational study enrolled 112 patients undergoing URSL for upper and mid-ureteric calculi at a single tertiary care centre between April 2024 and October 2025. A residual stone fragment was defined as any fragment >2 mm on non-contrast CT of the kidneys, ureters, and bladder (NCCT-KUB) at one month. Univariate analysis was performed using Student’s t-test, the Mann-Whitney U test, and the chi-square test or Fisher’s exact test. Receiver operating characteristic (ROC) curve analysis with Youden index optimization was performed for continuous predictors.

Results

Stone-free status was achieved in 102 of 112 patients (91.1%), while residual fragments were identified in 10 patients (8.9%). Hounsfield unit (HU) density was the strongest predictor of residual fragments (AUC, 0.920; 95% CI, 0.802-1.000; optimal cutoff, HU ≥1329; sensitivity, 100%; specificity, 82.4%; negative predictive value (NPV), 100%). All 10 residual cases occurred exclusively in patients with HU ≥1300; no patient with HU <1300 had a residual fragment. Stone size was a significant secondary predictor (13.4 ± 2.8 mm vs. 10.9 ± 2.8 mm; p = 0.009; AUC, 0.712). Stone location, anti-retropulsion device type, sex, age, and comorbidities were not significantly associated with residual fragment formation.

Conclusion

Preoperative HU density on NCCT-KUB is the strongest predictor of residual stone fragments after URSL, with a threshold of HU ≥1300 identifying all patients at risk with 100% sensitivity and an NPV of 100%. A stone size of ≥13 mm is a significant secondary predictor. Routine reporting of HU density may guide preoperative counselling, surgical planning, and the intensity of postoperative follow-up.

## Introduction

This manuscript reports the findings of a prospective observational cohort study and follows the Strengthening the Reporting of Observational Studies in Epidemiology (STROBE) guidelines for observational research. Urolithiasis is one of the most prevalent urological disorders globally, with ureteric calculi constituting a major proportion of stone disease. Ureteroscopic lithotripsy (URSL) is an established treatment for upper and mid-ureteric calculi, offering high stone-free rates with an established safety profile [1,2]. Advances in endoscopic design, instrumentation, and anti-retropulsion devices have substantially improved procedural outcomes over the past two decades.

Despite high overall stone-free rates, residual stone fragments remain a clinically important concern following URSL. Residual fragments may act as a nidus for stone recurrence, precipitate urinary tract infection, cause persistent obstructive symptoms, and necessitate auxiliary procedures, thereby increasing morbidity and healthcare costs [3]. The clinical significance of even small residual fragments is increasingly recognized in the contemporary literature [4].

Several preoperative and intraoperative factors have been proposed as determinants of incomplete stone clearance after URSL, including stone size, location, density, degree of impaction, and intraoperative retropulsion. Upper ureteric calculi carry a higher risk of proximal migration during lithotripsy. Anti-retropulsion devices, including the Stone Cone (Boston Scientific, Marlborough, MA, USA) and NTrap (Cook Urological, Bloomington, IN, USA), have been developed to mitigate this risk and improve outcomes.

Despite multiple published series on URSL outcomes, there remains a paucity of prospective data specifically evaluating predictors of residual fragments in upper and mid-ureteric calculi. Importantly, the majority of published studies examining Hounsfield unit (HU) density as a predictor have included heterogeneous cohorts treated with both pneumatic and laser lithotripsy, potentially obscuring the specific predictive contribution of stone density in the context of pneumatic lithotripsy, which is a commonly used modality in resource-limited healthcare settings globally.

The present study was therefore undertaken to prospectively evaluate the associations of preoperative clinical, stone-related, and operative variables with residual stone fragment formation following semi-rigid pneumatic URSL for upper and mid-ureteric calculi. It also aimed to assess the diagnostic performance of CT-derived quantitative imaging parameters, specifically HU density measured on preoperative non-contrast CT of the kidneys, ureters, and bladder (NCCT-KUB), using receiver operating characteristic (ROC) curve analysis; determine an optimal, clinically actionable HU threshold using the Youden index; and quantify the strength of the association between HU density and residual fragment formation using Firth’s penalized logistic regression, which is appropriate for datasets with a limited number of outcome events.

The study is explicitly exploratory and hypothesis-generating in design. It does not aim to establish independent predictors through conventional multivariable modelling but rather to characterize univariate associations and evaluate the diagnostic utility of a specific CT-derived imaging biomarker in a prospectively defined patient cohort.

## Materials and methods

Study design and setting

This prospective observational study was conducted in the Department of Urology and Renal Transplant at a single tertiary care teaching hospital between April 2024 and October 2025. This study constitutes a secondary analysis of prospectively collected data from a primary comparative study evaluating anti-retropulsion devices during URSL. Institutional Ethics Committee approval was obtained before study commencement, and written informed consent was obtained from all participants. The study was conducted in accordance with the Declaration of Helsinki. As this was a prospective observational study with no assignment of interventions by the investigators, clinical trial registration was not applicable.

Study population

Adults aged >18 years with radiologically confirmed upper or mid-ureteric calculi who were scheduled for URSL were eligible for inclusion. The exclusion criteria were pregnancy, active UTI at the time of surgery, congenital ureteral anomalies, prior ipsilateral ureteric surgery, and incomplete follow-up at one month. A total of 112 patients were enrolled during the study period.

Operative procedure

All procedures were performed under spinal or general anaesthesia using a semi-rigid ureteroscope with pneumatic lithotripsy. An anti-retropulsion device, either the Stone Cone or NTrap, was deployed before fragmentation in all patients. All procedures were performed or directly supervised by a single consultant urologist with more than 10 years of experience in endourology, ensuring procedural consistency throughout the study period. Semi-rigid ureteroscopy was performed using a 6/7.5 Fr ureteroscope. Pneumatic lithotripsy was delivered using a Swiss LithoClast pneumatic lithotripter (EMS, Nyon, Switzerland) with standard ballistic probe settings. Fragmentation was continued until all visible stone fragments were reduced to less than 2 mm, as assessed endoscopically. Irrigation was performed using normal saline at room temperature with gravity-dependent flow. Following complete lithotripsy, stone clearance was confirmed endoscopically, and a double-J stent was placed in all patients at the conclusion of the procedure.

Outcome assessment

**Figure 1:**
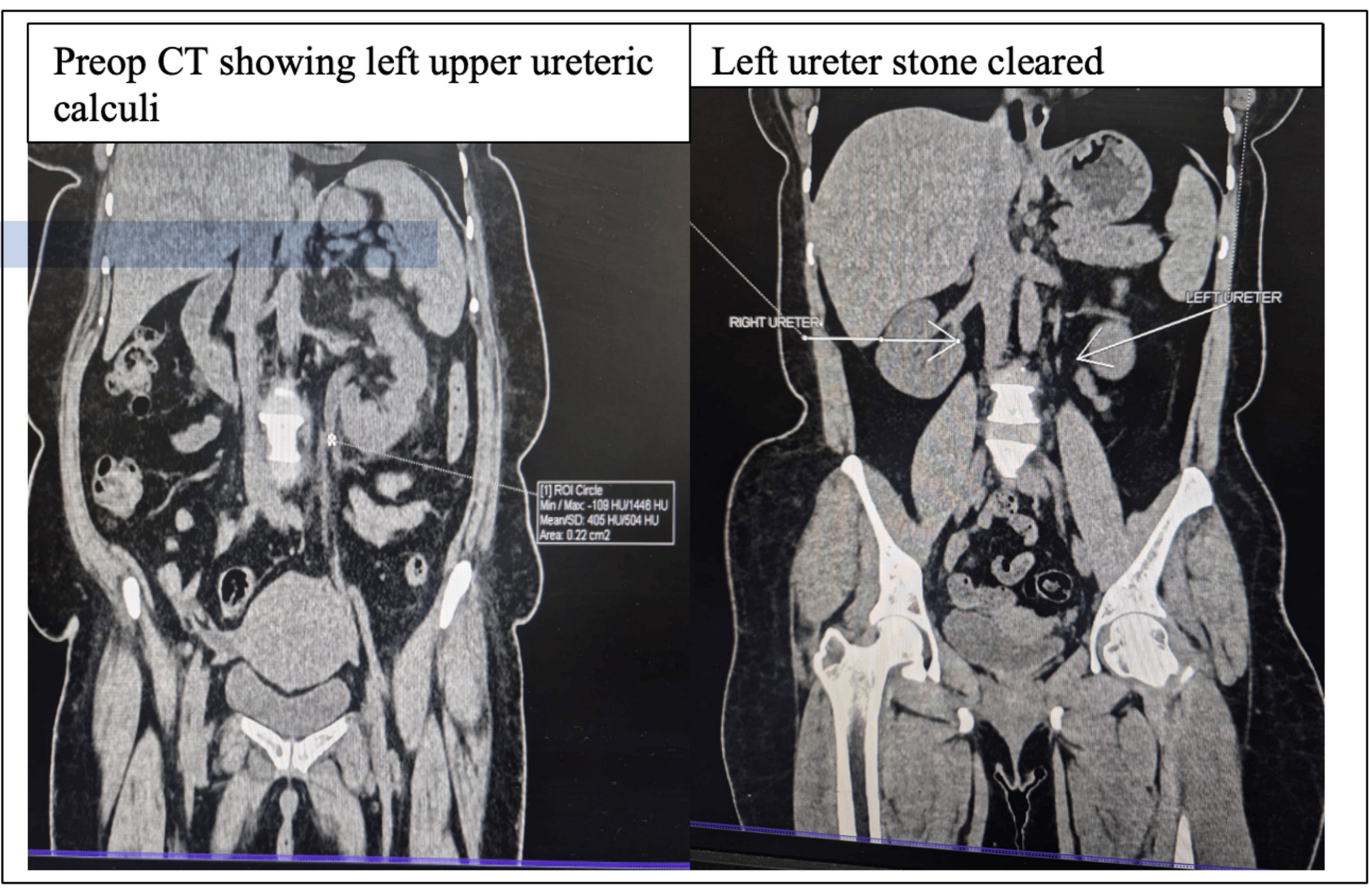
Low-density ureteric calculus. Preoperative coronal non-contrast computed tomography (NCCT) image demonstrating a low-density ureteric calculus with a circular region of interest (ROI) measurement (mean Hounsfield unit (HU) value, 405; area, 0.22 cm²). This patient achieved complete stone clearance at one month.

**Figure 2:**
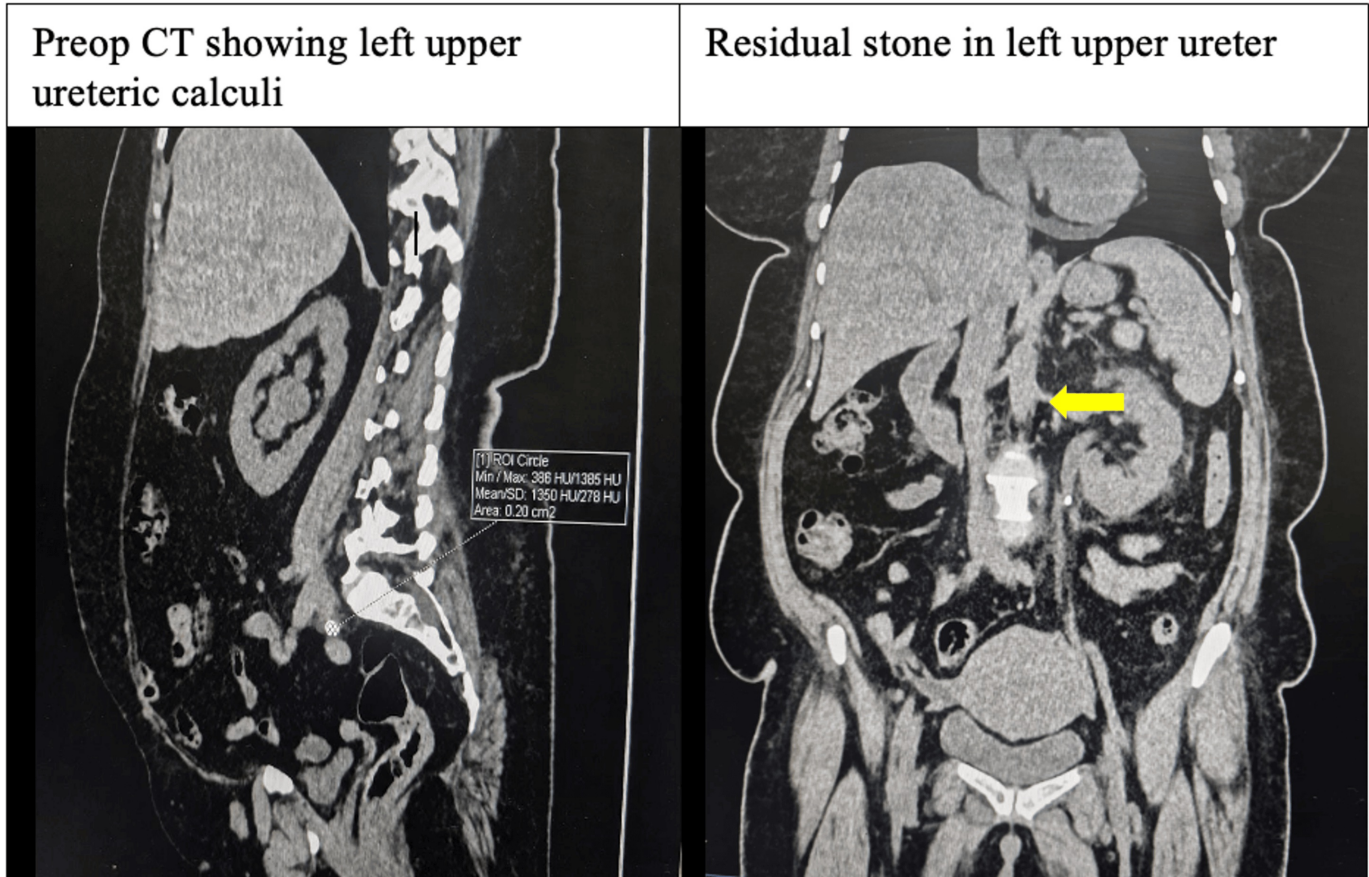
High-density upper ureteric calculus. Preoperative non-contrast computed tomography (NCCT) image demonstrating a high-density upper ureteric calculus with a region of interest (ROI) measurement (mean Hounsfield unit (HU) value, 1,350; maximum HU value, 1,385; area, 0.20 cm²). This patient had a residual stone fragment at the one-month follow-up.

All patients underwent NCCT-KUB at one month postoperatively. A residual stone was defined as the presence of any residual fragment measuring >2 mm on the one-month NCCT-KUB. The stone-free rate (SFR) was the primary outcome measure. Surgical complications were graded using the Clavien-Dindo classification. All NCCT-KUB examinations were performed using a GE Medical Systems Revolution ACTs multidetector CT scanner (GE Healthcare, Chicago, IL, USA) with standardized acquisition parameters: tube voltage of 120 kVp, automatic tube current modulation, slice thickness of 3 mm, pitch of 1.375, and a standard RFMT reconstruction kernel. Hounsfield unit measurements were obtained by placing a circular region of interest (ROI) of approximately 0.20-0.25 cm² within the largest area of the calculus, positioned centrally to minimize partial-volume averaging effects. Mean HU values were recorded as the representative stone-density measurement. All measurements were performed by a single trained radiologist with five years of experience in uroradiology who was blinded to the clinical and operative outcomes. The measurements were reviewed by a second radiologist, and consensus was reached for discrepant cases with a difference of >50 HU. Formal interobserver reproducibility analysis was not performed as a prospective component of the study, which represents a limitation. Representative preoperative NCCT-KUB images demonstrating the ROI measurement technique and postoperative images demonstrating residual-fragment and stone-free outcomes are provided in Figures 1-2.

Statistical analysis

Statistical analysis was performed using IBM SPSS Statistics, version 26.0 (IBM Corp., Armonk, NY, USA). Continuous variables were expressed as mean ± SD or median with IQR, as appropriate, and categorical variables were expressed as frequencies and percentages, n (%). Normally distributed continuous variables were compared between groups using Student’s independent-samples t-test and reported as t-statistics. Non-normally distributed continuous variables were compared using the Mann-Whitney U test and reported as U-statistics. Categorical variables were compared using the chi-square test (χ²) or Fisher’s exact test when cell counts were <5. All tests were two-tailed, and statistical significance was set at p < 0.05.

ROC curve analysis was performed for continuous predictors, with the AUC and 95% CI calculated using the Hanley-McNeil method. Optimal cutoff values were determined using the Youden index (sensitivity + specificity - 1).

Given the limited number of outcome events (n = 10), standard multivariable logistic regression was not considered appropriate because the events-per-variable ratio was <10. Firth’s penalized logistic regression was therefore used to assess the independent associations of significant univariate predictors with residual fragment formation, while correcting for small-sample bias and near-perfect separation. Models were constructed for HU density alone, stone size alone, and a combined model incorporating both variables simultaneously. Bootstrap internal validation with 1,000 iterations was performed to assess model optimism and internal validity following the method of Harrell et al. Model calibration was assessed using the Hosmer-Lemeshow goodness-of-fit test with five groups.

## Results

Study population and outcomes

A total of 112 patients (mean age, 39.4 ± 12.2 years; range, 21-74 years; 86 males, 76.8%) were enrolled. Upper ureteric calculi were present in 78 patients (69.6%), and mid-ureteric calculi were present in 34 patients (30.4%). Anti-retropulsion devices were equally distributed, with 56 patients each in the NTrap and Stone Cone groups. Residual stone fragments (>2 mm on NCCT-KUB at one month) were identified in 10 patients (8.9%), while 102 patients (91.1%) achieved complete stone clearance.

Demographic and clinical variables

Baseline demographic and clinical characteristics are presented in Table 1. Age (t = -0.277, p = 0.782), sex (χ² = 2.044, p = 0.153), diabetes mellitus (χ² = 0.375, p = 0.540), hypertension (χ² = 2.044, p = 0.153), preoperative DJ stenting (χ² = 0.069, p = 0.793), and prior urological procedures (p = 0.931) were not significantly associated with residual fragment formation.

**Table 1:** Baseline demographic and clinical characteristics of patients with and without residual fragments after ureteroscopic lithotripsy. Data are presented as n (%) for categorical variables and mean ± SD for continuous variables. Comparisons were performed using Student’s independent-samples t-test for age and the chi-square test (χ²) or Fisher’s exact test (*) for categorical variables when the expected cell count was <5. Statistical significance was set at p < 0.05. DJ: Double-J stent.

Variable	Residual fragments (n = 10), n (%)	Stone-free (n = 102), n (%)	Test statistic	p-value
Age, years, mean ± SD	38.4 ± 11.3	39.5 ± 12.4	t = -0.277	0.782
Sex, male	10 (100.0)	76 (74.5)	χ² = 2.044	0.153
Diabetes mellitus	0 (0.0)	12 (11.8)	χ² = 0.375	0.54
Hypertension	0 (0.0)	26 (25.5)	χ² = 2.044	0.153
Preoperative DJ stenting	8 (80.0)	72 (70.6)	χ² = 0.069	0.793
Prior urological procedure	4 (40.0)	34 (33.3)	χ² = 0.390*	0.931

Stone characteristics

Stone-related variables are summarized in Table 2. Patients with residual fragments had significantly larger stones than stone-free patients (13.4 ± 2.8 mm vs. 10.9 ± 2.8 mm; p = 0.009). HU density on preoperative NCCT-KUB was markedly and significantly higher in the residual-fragment group than in the stone-free group (1,506 ± 162 HU vs. 981 ± 298 HU; p < 0.001). Stone location (χ² = 0.112, p = 0.738) and multiplicity of calculi (p = 0.802) were not significantly associated with residual fragment formation. Notably, all 10 residual cases (100%) occurred exclusively among patients with HU ≥1,300 (χ² = 28.695, p < 0.001), and no patient with HU <1,300 had a residual fragment.

**Table 2:** Stone characteristics and univariate analysis of predictors of residual fragment formation. Data are presented as mean ± SD for continuous variables and n (%) for categorical variables. Comparisons were performed using Student’s independent-samples t-test for stone size and HU density and the chi-square test (χ²) or Fisher’s exact test (*) for categorical variables. Statistical significance was set at p < 0.05. HU: Hounsfield units; NCCT-KUB: Non-contrast computed tomography of the kidneys, ureters, and bladder.

Variable	Residual fragments (n = 10)	Stone-free (n = 102)	Test statistic	p-value
Stone size, mm, mean ± SD	13.4 ± 2.8	10.9 ± 2.8	t = 2.678	0.009
Stone density, HU, mean ± SD	1506 ± 162	981 ± 298	t = 5.477	<0.001
Stone location, upper ureter, n (%)	6 (60.0)	72 (70.6)	χ² = 0.112	0.738
Multiple calculi, n (%)	2 (20.0)	12 (11.8)	χ² = 0.063*	0.802
HU ≥1,300, n (%)	10 (100.0)	18 (17.6)	χ² = 28.695	<0.001

Operative parameters and intraoperative outcomes

Operative parameters are presented in Table 3. Operative duration showed a nonsignificant trend toward a longer duration in the residual-fragment group (66.0 ± 23.7 minutes vs. 55.0 ± 19.0 minutes; t = 1.706, p = 0.091). Anti-retropulsion device type was not significantly associated with residual stone formation (χ² = 2.745, p = 0.098); stone-free rates were 48 of 56 patients (85.7%) in the NTrap group and 54 of 56 patients (96.4%) in the Stone Cone group. Intraoperative haemorrhage was significantly more frequent in the residual-fragment group (4 (40.0%) vs. 4 (3.9%); χ² = 12.847, p < 0.001). The surgeon difficulty score was significantly higher in the residual-fragment group (median, 4 (IQR, 4-4) vs. 2 (IQR, 2-3); U = 944.0, p < 0.001). The duration of hospital stay was significantly longer among patients with residual fragments (3.8 ± 1.0 days vs. 2.2 ± 0.5 days; t = 8.761, p < 0.001). Postoperative auxiliary procedures were required in all 10 patients (100%) with confirmed residual fragments, compared with none of the stone-free patients (0 (0.0%); Fisher’s exact test, p < 0.001).

**Table 3:** Operative parameters, intraoperative findings, and postoperative course. Data are presented as n (%) for categorical variables, mean ± SD for normally distributed continuous variables, and median (IQR) for non-normally distributed variables. Comparisons were performed using Student’s independent-samples t-test for operative time and duration of hospital stay, the chi-square test (χ²) for categorical variables, the Mann-Whitney U test for surgeon difficulty score, and Fisher’s exact test (*) when the expected cell count was <5. Statistical significance was set at p < 0.05.

Variable	Residual fragments (n = 10)	Stone-free (n = 102)	Test statistic	p-value
Operative time, min, mean ± SD	66.0 ± 23.7	55.0 ± 19.0	t = 1.706	0.091
Device, NTrap, n (%)	8 (80.0)	48 (47.1)	χ² = 2.745	0.098
Ureteral injury, n (%)	2 (20.0)	4 (3.9)	Fisher's exact*	0.156
Intraoperative haemorrhage, n (%)	4 (40.0)	4 (3.9)	χ² = 12.847	<0.001
Surgeon difficulty score, median (IQR)	4 (4-4)	2 (2-3)	U = 944.0	<0.001
Postoperative auxiliary procedure, n (%)	10 (100.0)	0 (0.0)	Fisher's exact*	<0.001
Duration of hospital stay, days, mean ± SD	3.8 ± 1.0	2.2 ± 0.5	t = 8.761	<0.001

ROC curve analysis

The results of the ROC curve analysis are presented in Tables 4-5. HU density demonstrated excellent discriminative performance (AUC, 0.920; 95% CI, 0.802-1.000). The optimal cutoff derived using the Youden index was HU ≥1,329 (sensitivity, 100%; specificity, 82.4%; NPV, 100%). The clinically practical threshold of HU ≥1,300 also achieved 100% sensitivity, with no false negatives (Table 5).

**Table 4:** Receiver operating characteristic (ROC) curve analysis: discriminative performance of continuous predictors for residual fragment formation. AUC: Area under the curve; HU: Hounsfield unit. The 95% CIs were calculated using the Hanley-McNeil method. AUC values were interpreted as follows: ≥0.90, excellent; 0.80-0.89, good; 0.70-0.79, acceptable; and <0.70, poor.

Variable	AUC	95% CI	Sensitivity	Specificity
Stone density, HU (cutoff ≥1,329)	0.92	0.802-1.000	100.00%	82.40%
Surgeon difficulty score (cutoff ≥4)	0.926	0.811-1.000	80.00%	91.20%
Stone size (cutoff ≥13 mm)	0.712	0.525-0.898	60.00%	82.40%
Operative time	0.647	0.455-0.840	-	-

**Table 5:** Hounsfield unit density threshold analysis: sensitivity, specificity, and predictive values at incremental cutoff points. ^†^Indicates the optimal threshold determined using the Youden index. PPV: Positive predictive value; NPV: Negative predictive value; TP: True positive; FP: False positive; TN: True negative; FN: False negative. The Youden index was calculated as sensitivity + specificity - 1.

HU threshold	Sensitivity	Specificity	PPV	NPV	TP/FP/TN/FN	Youden index
≥900	100.00%	35.30%	13.20%	100.00%	10/66/36/0	0.353
≥1,000	100.00%	52.90%	17.20%	100.00%	10/48/54/0	0.529
≥1,100	100.00%	70.60%	25.00%	100.00%	10/30/72/0	0.706
≥1,200	100.00%	76.50%	29.40%	100.00%	10/24/78/0	0.765
≥1,300†	100.00%	82.40%	35.70%	100.00%	10/18/84/0	0.824
≥1,400	80.00%	84.30%	33.30%	97.70%	8/16/86/2	0.643

**Figure 3:**
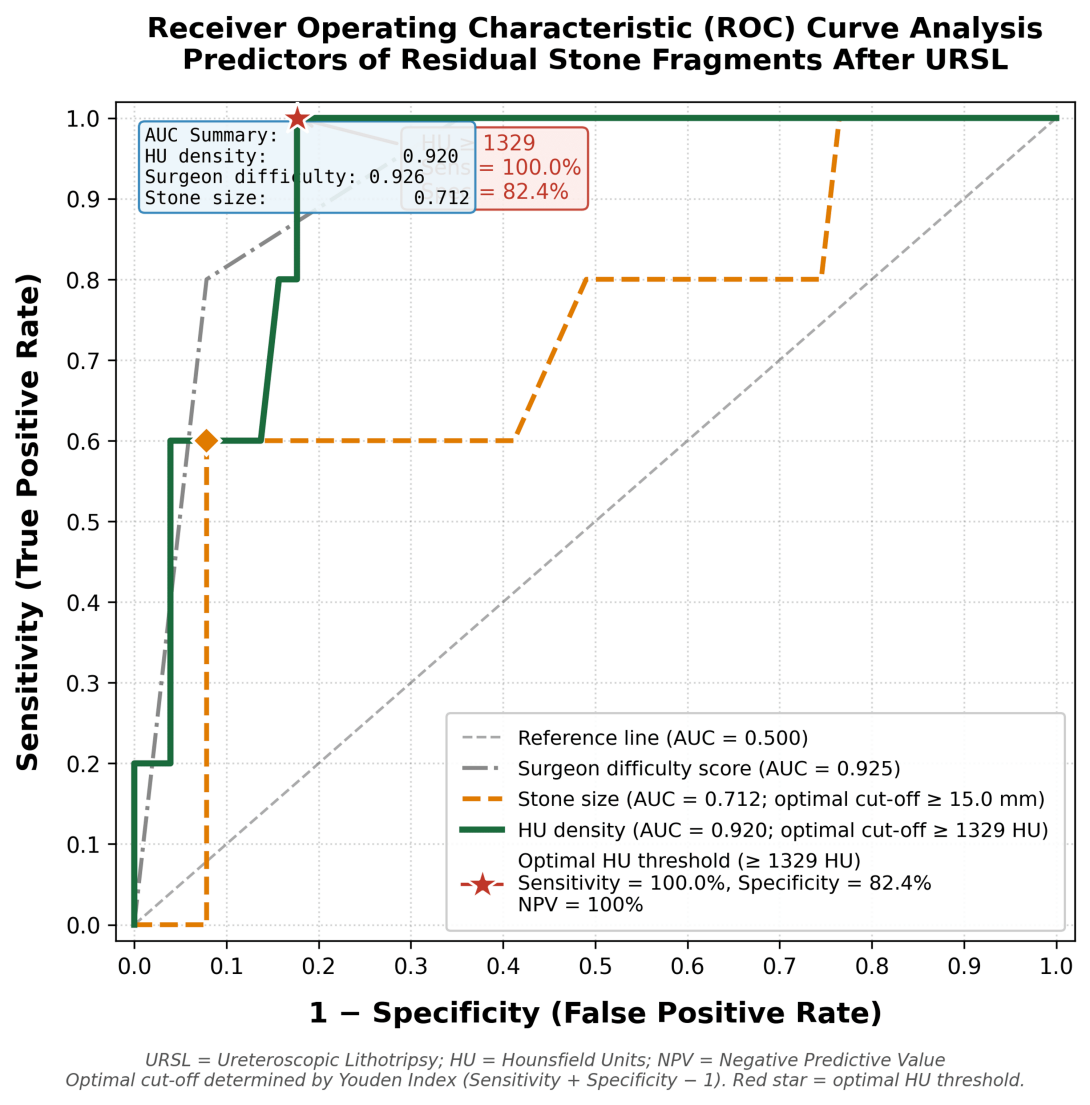
Receiver operating characteristic (ROC) curves for continuous predictors of residual stone fragment formation. HU density demonstrated excellent discrimination (AUC = 0.920; optimal cutoff, ≥1,329 HU; sensitivity, 100%; specificity, 82.4%; NPV, 100%). Stone size showed acceptable discrimination (AUC = 0.712; optimal cutoff, ≥13 mm). The surgeon difficulty score demonstrated excellent discrimination (AUC = 0.926) but represents an intraoperative variable. The red star indicates the optimal Youden index threshold for HU density. AUC: Area under the curve; HU: Hounsfield units; NPV: Negative predictive value.

The surgeon difficulty score had a comparable AUC (0.926; 95% CI, 0.811-1.000) but represents an intraoperative variable that is unavailable for preoperative prediction. Stone size showed acceptable discrimination (AUC, 0.712; 95% CI, 0.525-0.898; optimal cutoff, ≥13 mm; sensitivity, 60%), missing 4 of the 10 residual cases. Operative time was a poor predictor (AUC, 0.647; 95% CI, 0.455-0.840) (Figure 3).

Firth’s penalized logistic regression

Given the limited number of outcome events, Firth’s penalized logistic regression was performed. On univariable Firth regression, each 100-HU increase in stone density was associated with significantly higher odds of residual fragment formation (OR, 1.973; 95% CI, 1.360-2.864; p < 0.001). Stone size was also a significant univariable predictor (OR, 1.295 per mm; 95% CI, 1.054-1.591; p = 0.014). In the combined Firth regression model incorporating both HU density and stone size simultaneously, HU density retained a significant independent association (OR, 7.265 per SD; 95% CI, 2.273-23.221; p = 0.001), while stone size did not remain significant after adjustment (OR, 1.691; 95% CI, 0.765-3.737; p = 0.194). These results indicate that HU density is not simply a surrogate for stone size. The odds ratio for HU ≥1,300 as a binary predictor was 95.9 (95% CI, 5.3-1,744.7; p = 0.002), with the wide CI reflecting the limited number of outcome events (Table 6) [5].

**Table 6:** Firth’s penalized logistic regression analysis. The SD was 324 HU for stone density and 2.9 mm for stone size. Firth’s penalized logistic regression was used because of the limited number of outcome events (n = 10) and near-perfect separation. The third and fourth rows present the results of the combined multivariable model. HU: Hounsfield units.

Variable	Model	OR	95% CI	p-value	Significance
Stone density (per 100-HU increase)	Univariable	1.973	1.360-2.864	<0.001	Significant
Stone size (per 1-mm increase)	Univariable	1.295	1.054-1.591	0.014	Significant
Stone density (per SD increase of 324 HU)	Combined multivariable	7.265	2.273-23.221	0.001	Significant
Stone size (per SD increase of 2.9 mm)	Combined multivariable	1.691	0.765-3.737	0.194	Not significant
Stone density ≥1,300 HU	Univariable	95.919	5.274-1744.7	0.002	Significant

Bootstrap internal validation

Bootstrap resampling validation with 1,000 iterations was performed to assess model optimism. For the HU-density model, the apparent AUC of 0.920 demonstrated negligible optimism (mean optimism = 0.0002), yielding an optimism-corrected AUC of 0.919 (bootstrap 95% CI, 0.852-0.974). For the combined HU-density and stone-size model, the optimism-corrected AUC was 0.924 (bootstrap 95% CI, 0.864-0.983; mean optimism = 0.005). Hosmer-Lemeshow goodness-of-fit testing indicated good calibration for both models (HU-density model: χ² = 2.988, p = 0.393; combined model: χ² = 0.969, p = 0.809). The near-zero optimism values indicate that the diagnostic performance estimates were not materially inflated by overfitting within this dataset.

Complications

Postoperative complications occurred in 22 patients (19.6%): Clavien-Dindo grade I in 8 patients (7.1%), grade II in 10 patients (8.9%), and grade III in four patients (3.6%). No major ureteral injuries or life-threatening complications were encountered. Minor mucosal injury and transient pyrexia were the most common complications [6,7].

## Discussion

This prospective study demonstrated an overall stone-free rate (SFR) of 91.1% following URSL for upper and mid-ureteric calculi, consistent with contemporary published series [8-10]. The principal finding was that stone density, measured in HU, was strongly associated with residual fragment formation, with an HU threshold of ≥1,300 identifying all residual cases in this cohort. In Firth’s penalized logistic regression, HU density retained an independent association after adjustment for stone size (OR, 7.265; p = 0.001), and bootstrap validation demonstrated negligible overfitting (optimism, 0.0002). These findings are exploratory and hypothesis-generating and do not constitute definitive clinical recommendations.

Stone density in HU reflects the mineral composition and crystalline compactness of the calculus. Higher HU values indicate harder stones that may be more resistant to pneumatic fragmentation, thereby increasing the likelihood of incomplete pulverization [11-13]. The exclusive use of pneumatic lithotripsy in this study is an important mechanistic consideration. Unlike laser lithotripsy, in which pulse energy and fragmentation mode can be adjusted intraoperatively to compensate for stone hardness, pneumatic lithotripsy delivers fixed ballistic energy. This makes stone density a particularly relevant determinant of fragmentation success in the pneumatic context and may explain why the association between HU density and outcomes emerged so clearly in this specific operative setting. Whether the same threshold applies to laser lithotripsy cohorts, in which operators can partially overcome stone resistance through technical adjustments, requires separate prospective evaluation.

Stone size was also a significant predictor (t = 2.678, p = 0.009), with a mean stone size of 13.4 ± 2.8 mm in the residual-fragment group compared with 10.9 ± 2.8 mm in stone-free patients. However, with an AUC of only 0.712 and a sensitivity of 60% at the optimal cutoff of ≥13 mm, stone size alone was an insufficient discriminator.

The present findings are contextualized within the published multicentre literature. Rippel CA et al., in a landmark two-centre retrospective study of 265 ureteroscopic procedures with postoperative CT follow-up, reported a residual fragment rate of 38% and identified stone size as the sole independent predictor in multivariable analysis; HU density was not evaluated [14]. The higher SFR in our cohort (91.1%) likely reflects patient selection, as only ureteric stones were included, as well as operative standardization and consistent use of anti-retropulsion devices. Kim JW et al. developed a CT-based URSL prediction model in 237 patients (AUC, 0.825) but found that HU was not an independent predictor in standard regression, a finding potentially attributable to events-per-variable constraints that Firth’s method, which is specifically designed for rare-event data, may help address [15]. Lopategui DM et al., in a recent multicentre real-world dataset, did not evaluate HU as a primary predictor, representing the gap that the present study specifically addresses [16].

Contrary to expectations, stone location (upper vs. mid ureter; p = 0.738) was not significantly associated with residual fragment formation. This may reflect the systematic use of anti-retropulsion devices in all cases, which could have reduced the location-dependent disadvantage of proximal migration. The SFRs in the NTrap and Stone Cone groups were 85.7% and 96.4%, respectively (p = 0.098), which is consistent with earlier comparative data [17-19].

Intraoperative haemorrhage (χ² = 12.847, p < 0.001) and surgeon difficulty score (U = 944.0, p < 0.001) were significantly associated with residual fragments, likely representing consequences of high-density, resistant stones rather than independent causal predictors. These intraoperative findings reinforce the predictive importance of preoperative HU density.

This study has several important limitations that must be acknowledged. First, the small number of outcome events (n = 10, 8.9%) represents the principal statistical limitation, precluding standard multivariable analysis and rendering all diagnostic performance estimates exploratory. Second, although Firth’s penalized regression and bootstrap validation provide reassurance regarding internal robustness, they do not substitute for external validation in an independent multicentre cohort, which is the essential next step. Third, the exclusive use of pneumatic lithotripsy limits generalizability to laser lithotripsy settings. Fourth, the single-centre design restricts generalizability across institutions, patient populations, and imaging protocols. Fifth, HU values may vary across CT scanner models and acquisition protocols; therefore, the proposed threshold should not be applied in other settings without scanner-specific validation. Sixth, formal interobserver and intraobserver reproducibility of HU measurements was not assessed. Seventh, the surgeon difficulty score is a subjective intraoperative measure. Multicentre prospective studies with larger numbers of outcome events are warranted to validate the HU threshold identified herein.

## Conclusions

Management of upper and mid-ureteric calculi with URSL continues to produce excellent outcomes, and the overall stone-free rate of 91.1% observed in this prospective cohort reflects the standard of care achievable through a systematic and standardized surgical approach.

Beyond the surgical outcomes, this study offers a practically useful finding: a simple, preoperatively available value that appears to meaningfully stratify patients according to their likelihood of achieving complete stone clearance. Stone density, measured in Hounsfield units on routine preoperative CT, emerged as the strongest predictor associated with residual fragment formation, with a threshold of approximately 1,300 HU identifying every patient who subsequently developed a residual fragment. Importantly, this association remained significant even after statistical adjustment for stone size, suggesting that stone density provides predictive information beyond that offered by stone dimensions alone.

The clinical promise of this finding lies in its simplicity. The HU value can be obtained from routine preoperative CT imaging, requiring no additional investigation, added cost, or change to the operative workflow. Incorporating it into the preoperative assessment may allow urologists to counsel patients more accurately, plan follow-up more appropriately, and set realistic expectations before surgery.
